# Feasibility of Kinect-Based Games for Balance Rehabilitation: A Case Study

**DOI:** 10.1155/2018/7574860

**Published:** 2018-07-09

**Authors:** Ines Ayed, Adel Ghazel, Antoni Jaume-i-Capó, Gabriel Moya-Alcover, Javier Varona, Pau Martínez-Bueso

**Affiliations:** ^1^GresCom Lab, Ecole Supérieure des Communications de Tunis, Université de Carthage, Tunis, Tunisia; ^2^Unitat de Gràfics, Visió per Computador i Intel·ligéncia Artificial, Department de Ciències Matemàtiques i Informàtica, Universitat de les Illes Balears, Palma, Spain; ^3^Research Group on Evidence, Lifestyles and Health, Department of Nursing and Physiotherapy, University of the Balearic Islands, Palma, Spain

## Abstract

We aimed at determining the effects of prototype games on older adults attending a rehabilitation program in an elderly house in this work. We conducted an initial case study where two participants underwent a 5-week intervention. Feasibility was assessed by examining recruitment, adherence, and safety. The Tinetti balance test was used as pretest and posttest assessments. Results show that adherence was very high and no adverse effects were registered during the sessions. The included participants also reported enjoyment during the playtime and exhibited improvements in Tinetti scores. The findings suggest that game-based rehabilitation can be useful for improving balance in elderly people and can be incorporated in a fall prevention program.

## 1. Introduction

Falls are prevalent among elderly people. More than a third of people aged 65 years and over fall at least once per year [1, 2]. Risk of falls is even higher within institutional residents than among community dwelling older people, with an incidence of 1.5 falls per bed per year [3, 4]. In fact, housing type was determined as one of the extrinsic factors predisposing to risk of falls and fractures [5]. However, previous falls and deficits in gait and balance have been identified as significant risk factors as they were highly correlated with falling [2, 3, 6]. In fact, falls are the leading cause of injury, deaths, and hospital admissions for traumatic injuries among people aged 65 and older [1]. Adopting preventive measures and implementing effective fall prevention programs and interventions help us to reduce the risk of falls and minimize the substantial social and healthcare costs induced by falls [7, 8]. Moreover, decreasing fall-related injury incidences could significantly improve quality of life of older adults and their caregivers [9].

Most of the fall prevention programs include motor rehabilitation with exercise interventions. A fall prevention meta-analysis showed that exercise programs that challenged balance were associated with significant reductions in fall rates [8].

Recently, serious games have been proven to be effective in motor rehabilitation [10]. Serious games are games designed for a primary purpose different from the purpose of pure entertainment; the cognitive and motor activities required by these games attract the attention of users [11], and this helps to distract them from the task [12, 13]. Healthcare-related serious games can be deployed in treatment, recovery, and rehabilitation. It is demonstrated that these games are highly promising in maintaining motor skills since they help us to motivate the patients to adhere to repetitive and intensive therapy sessions [14]. In long-term rehabilitation programs, patient's demotivation is frequent because of the repetition and boredom of rehabilitative activities, which may cause the lack of focus or the abandonment of the program, and consequently the loss of the benefit and effectiveness of the therapy.

Elderly population was the target of many studies that examined the games' effectiveness in balance training [15–17]. Though much more research in this area is needed, published findings show that video games can be remedies for minimizing risk of falls [18, 19]. In addition, vision-based systems are proven to be suitable for elderly people as they are motivating and noninvasive [20–23]. While exist many vision-based interfaces, Microsoft Kinect gained much interest in rehabilitation in recent years by offering a natural human computer interaction. Kinect is a low-cost RGBD sensor that captures colour and depth data providing full-body tracking and gesture recognition. It enables the user to interact with the game without the need to use a controller device.

Several studies have investigated the feasibility of using Kinect with elderly participants employing commercial games to improve balance and gait [24, 25]. Nevertheless, usability tests conducted in [26] show that some commercially available games are not suitable for therapeutic purposes and provide negative auditory and visual feedback during game tasks. Thus many research studies proposed prototype games specified for rehabilitation and addressed to defined target groups. For instance, Hoda et al. [27] aimed at improving the movement and control of upper limbs of elderly stroke patients; Ofli et al. [28] suggested a set of exercises for the improvement of balance, flexibility, strength, and endurance of independently living older adults; and authors in [29] investigated the feasibility of a rehabilitation game for dynamic postural control of people with Parkinson's disease. Although there is evidence that serious games improve balance of elderly people in general, there is a lack of evidence about their effects, under the supervision of physiotherapists, on institutionalized older adults and their deployment in elderly houses and institutions. Accordingly, this paper is an exploratory and descriptive case study [30] about the impact that the games could have in the development of future rehabilitation games for the fall prevention programs for elderly people using vision-based technologies. Such works are preliminary studies that help in the design and preparation of randomised clinical trials [31]. They tend to describe the feasibility and potential effectiveness of the system proposed at a lower cost and shorter time.

The aim of this work was to investigate the feasibility and effectiveness of prototype Kinect-based serious games that focus on postural control and balance rehabilitation in elderly people. In the study, we used Microsoft Kinect as the vision-based interface, and we hypothesised that the games would have a positive impact on the participants attending the elderly house.

## 2. Materials and Methods

This section explains how we assessed the feasibility of Kinect-based games for balance rehabilitation in the elderly house. First, we present the serious game implementation. Second, we describe the case trial design.

The serious games developed for this study try to imitate exercises included in traditional physical therapy, such as reaching in different directions, small and large lateral steps, weight shifting to both sides, neck movements (flexion, extension, lateral flexion, and rotation), shoulder movements (flexion, extension, adduction and abduction), trunk movements (flexion, extension, lateral flexion, and rotation), knee movements (flexion and extension), and hip movements (flexion, extension, rotation, adduction and abduction) [32–34]. These, however, had the added value that the immersive virtual environment tried to hook the patient to the point of not focusing on the fact of being in a rehabilitation session. This rehabilitation method using serious games is proposed for patients with risk of falls in order to improve their balance.

### 2.1. Game Design and Implementation

A series of games focusing on the physical rehabilitation of the balance and postural control of elderly people were designed and developed according to the requirements and indications from physiotherapists [35]. In the design process, we adopted the guidelines for developing rehabilitative games in order to offer engaging exercises that predispose motivation, feedback, and game monitoring [36]. Safety of the games is also very important; thus, game parameters were configured to allow the physiotherapist to adapt the exercises for each user according to his needs and preferences. The developed games are the following:

- *Reach game*: Here, the users had to move their centre of mass (COM) in order to reach with their hands one of the five balls located on their user plan. Once the user touched a ball, it disappeared and reappeared after a determined time set by the physiotherapist according to the user's speed. The two symmetric items added previously on the level of hips were deleted because the participants found it very difficult to do weight shifting movements. To further adapt the games for the participants recruited, the balls were resized and their colours were set to black and red over a simple white background. Data like user id, session date, play duration, and distance from the sensor were automatically recorded and saved in an excel document for any later check or use.
- *HitIt*: In this game, soccer balls fall randomly within the same plan of the user. To hit them, the user needs to make lateral steps, and touch them with his head when they are at his level. The use of any other part of the body, except the head, goes unperceived during the game play. The game can be also played in a seated position. The user has to make lateral movements of the trunk to be able to touch the elements with his head.
- *WatchOut*: Here, subjects have to move laterally in order to escape falling eggs. Falling items fall randomly within the same plan of the user with adaptable falling rate and speed.

In all games, a background with a solid colour has been defined to keep the user focused on the game.

#### 2.2. Trial Design

We present a case study to describe in detail a patient's episode of care and to assess a new initiative in a health service [30, 37]. We undertook an intrinsic case study to investigate the feasibility and effectiveness of prototype Kinect-based serious games on postural control and balance rehabilitation in a group of elderly people. And this was conducted into the specific context of an elderly house in Tunisia, where demographic changes have decreased fertility due to aging population. Persons aged more than 65 in the country constituted 8% of the total population in 2015; this percentage is considered the highest in Arab region. In 2050, it is predicted to reach 20 percent of the population [38].

#### 2.2.1. Participants

Participants were chosen by a physiotherapist to participate in this study and were recruited in an elderly house. The inclusion criteria were as follows: (1) aged over 55 years; (2) ability to understand, learn, and follow simple instructions; and (3) voluntary agreement to participate in the clinical study. The exclusion criteria were as follows: (1) severe cognitive deterioration; (2) profound bilateral hearing loss with the use of hearing aids; (3) hemiplegia, dementia, or Parkinson; (4) serious or uncontrolled epilepsy; and (5) serious or recurring medical complications.

Total population was screened for eligibility in the elderly house of Manouba in Tunisia: 120 older adults. Among them, only 32 met the inclusion and exclusion criteria. The research team made a request to participate in the intervention to all adults who met the inclusion criteria. 15 subjects accepted to participate in the study but only 8 showed up for a trial session that lasted between 10 and 15 min. Those who joined the trial session liked the games, but for different reasons, some of them could not adhere to the trial sessions (one got a job and others had time incompatibility with doctor visit or their time of hanging out of the elderly house), so the final case study sample included only 3 subjects. One of the participants dropped in the middle of the intervention as she left the elderly house, and 2 completed the intervention and the pre- and postassessments. Subjects who accepted to participate were illiterate and were screened for cognitive impairment using Arabic-Mini Mental State Examination (A-MMSE) [39]. An A-MMSE score higher than 20 suggested that both participants had no severe cognitive impairment as defined in the exclusion criteria.

For accessibility reasons, the serious game program was provided to users with different conditions (education, history of falls, cognitive impairment level, and balance problems). The two subjects included in this case study [40] met also the following criteria:

1. Subjects' capabilities were stable which means there would not be any evolvement in their capabilities during the study due to some pathology.
2. Users were attending intervention sessions according to the study schedule, so they were not exercising out of this time. This criterion provided homogeneity to the context of study.

The characteristics of the participants are presented in Table 1. An informed consent was signed before the intervention.

#### 2.2.2. Procedure

The intervention was conducted along a period of 5 weeks. Each participant underwent one 30 min session per day at a rate of 3 days a week. The rehabilitation program was divided as follows: 20 min was devoted for *Reach game*, 10 min for *HitIt*, and an extra 5 min was added for *WatchOut* game starting from session number 9. The duration of each game was set according to its understanding and its acceptability by the participants. A break time between 3 and 6 min was allowed each 5 min of play regarding the fatigue level.

**Figure 1:**
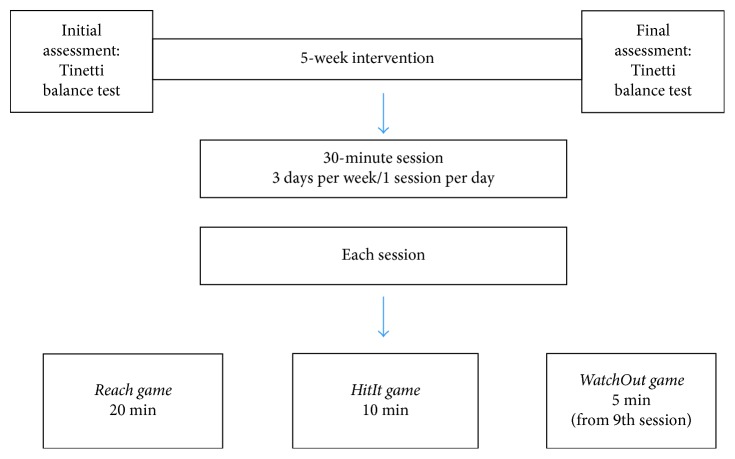
Intervention scheme.

Balance was assessed using the Tinetti balance test at the beginning (initial assessment) and at the end (final assessment) of the intervention. Adherence, game scores, and adverse events were noted along the intervention. The design of the clinical intervention is presented in Figure 1.

**Figure 2:**
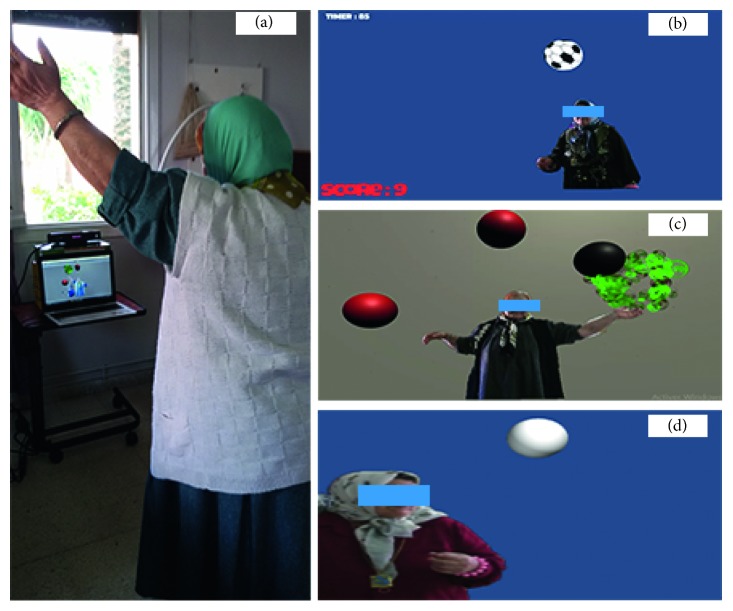
(a) System settings; (b) *HitIt* game; (c) *Reach game*; (d) *WatchOut* game.

The system setting consisted of a personal computer and Microsoft Xbox Kinect sensor (Figure 2). In the literature, a vision-based system that processes higher than 19 fps its response is considered real time [41]. All games were designed to process at least 25 fps, and this ensures a real-time interaction. The participants stood in front of the camera which detected their presence and allowed them interacting with virtual objects appearing on the screen. The Kinect camera was placed at a fixed height of 1 m and the average distance from the camera was 1.5 m. The games used were *HitIt* (Figure 2), *Reach game* (Figure 2), and *WatchOut* (Figure 2).

#### 2.2.3. Measurements

Gaming was assisted by an occupational therapist (OT) and monitored by the research team.

As stated before, all subjects were clinically evaluated prior to the intervention program and again at the conclusion to assess their balance with the Tinetti balance test.

The Tinetti test was performed by the OT who was not blinded to the intervention. Other measurements were registered in a case report form, by the research team or saved automatically by the system.

To assess the feasibility of using vision-based rehabilitation games for balance and postural control in the elderly house, the following outcome measurements were used:

- *Tinetti balance test:* The Tinetti assessment tool is a simple and easily administered test that measures a patient's gait and balance [42]. The test scoring is done on a three-point ordinal scale with a range of 0 to 2 where a score of 0 represents the most impairment, while 2 would represent independence of the patient. The individual scores are then combined to form three measures: an overall gait assessment score, an overall balance assessment score, and a gait and balance score. The maximum score for the gait component is 12 points. The maximum score for the balance component is 16 points. The maximum total score is 28 points. In general, patients who score below 19 are at a high risk for falls, while a score in the range of 19–24 indicates that the patient has a risk for falls [43].
- *Adherence:* The research team registered the number and length of sessions. Planned playing time and actual playing time were recorded by the system.
- *Game score:* The score of *HitIt* game was saved by the system at each session. Whenever the participant touched the ball with her head, one point was added to the score. The scores of the other games were not considered in this case since they did not truly reflect the progress of the participant. Besides, the *HitIt* game score was used to motivate the patients and create a kind of competitiveness between them.
- *Position of the patients and healthcare assistant:* The position of the participants as seated or standing and the location of the therapist with respect to the participants were noted during intervention sessions.
- *Adverse events:* Events such as falls, fatigue, or any safety incidents requiring medical attention were also documented. Participants were orally asked for feedback during each session for any further improvements or suggestions.

## 3. Results

We are interested in reporting the results of the two participants who completed the intervention and performed the pre- and postassessments.

### 3.1. Balance

According to the Tinetti test scores, both participants showed a similar trend. As shown in Table 2, there was an improvement of 4 points in the balance section score, while almost no difference was noted between pre- and postmeasures in the gait section. In the preassessment, Participant 1 had a total score of 15/28 (balance: 7/16; gait: 8/12) and Participant 2 had a total score of 14/28 (balance: 6/16; gait: 8/12). In the postassessment, Participant 1 had a total score of 19/28 (balance: 11/16; gait: 8/12) and Participant 2 had a total score of 17/28 (balance: 10/16; gait: 7/12).

### 3.2. Adherence

**Figure 3:**
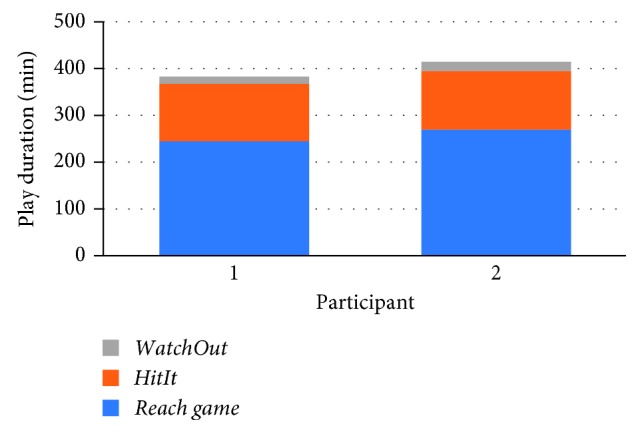
Time spent by each participant in each game.

Participants attended 86.6% of the sessions with an average 30 min length each. As depicted in Figure 3, Participant 1 had 363 min of a total playing time divided between *Reach game* (245 min), *HitIt* (123 min), and *WatchOut* (15 min). Similarly, Participant 2 spent around 415 min of a total playing time divided between *Reach game* (270 min), *HitIt* (125 min), and *WatchOut* (20 min).

### 3.3. Game Score

**Figure 4:**
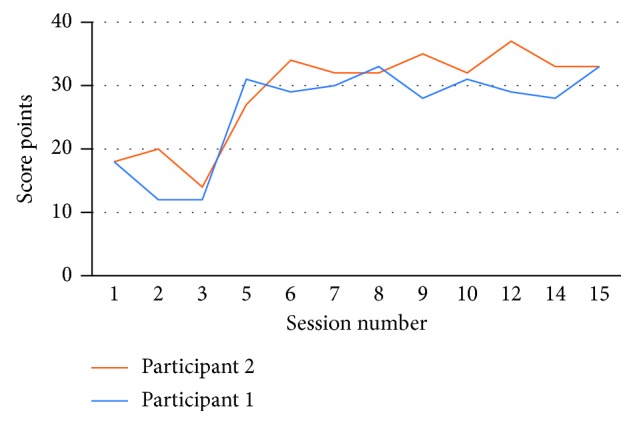
*HitIt* game score for the two participants along the experiment.

The game score improved over time, although there were no significant differences over time between the two participants as shown in Figure 4.

### 3.4. Position of the Participant and Healthcare Assistant

In all sessions, the participants were standing during playtime, and they sat on a chair during the breaks. As for the therapist, she stood behind the participants the first sessions as they expressed fear of fall and needed more help in doing lateral steps in *HitIt* and *WatchOut* games or reaching objects in *Reach game*. By the end, the participants could stand without any support and the assistant stood on their left or right side, between one and three meters away, with the exception of Participant 1 who had a relapse after the fall accident and needed more support for restoring her confidence again.

### 3.5. Adverse Events

Participant 1 fell during the intervention period; precisely after the ninth session, it was on a weekend day, not in the training day because she did not use her ambulatory aids that day. She returned to assist the training sessions just two days after the fall accident. At the beginning, she felt unsecure and needed more assistance as someone had to stand behind her for reassurance, but sooner she started to gain confidence again. Besides, it is worth mentioning that she got tired easily in the postfall period, usually both participants reported fatigue after 5 or 6 min of training, but she asked for a break after 2 or 3 min. No serious adverse events were reported from Participant 2.

## 4. Discussion

As far as we reviewed, it is a first case study conducted in a Tunisian elderly house using Microsoft Kinect and serious games for balance rehabilitation and postural control.

The findings show that the recruitment was very low, and this is due to the following. First, the recruitment period was short which partially explains the low participation between the elderly residents of the institution.

Second, exergaming was a new concept for many older adults. In fact, some refused to participate thinking it would be difficult for them to understand the games looking to the technology used, and others considered themselves too old to play. Consequently, we had to change the terminology used to stimulate them to participate at least to a trial session. We believe that using terms as “exercises” and “training” rather than “games” and “playing” may increment the recruitment rate.

Third, disliking exergames was also associated with low recruitment and high dropout rate as shown in another study suggesting that elderly people go for paper-based instructions to practice self-regulated conventional exercise instead of using computer-based exergames [44].

Nonetheless, the results related to the two participants were quite promising. Regarding the Tinetti balance test, both participants have shown an improvement of 4 points in the total score over the study period; the increase in the score was noticed especially in the balance section. The Tinetti test measures balance and gait, but our system focused particularly on balance; this justifies the absence of significant differences in the gait section as well. However, with this improvement, Participant 1 moved from high risk for falls range to the risk for falls range, while participant 2 is still at high risk for falls [43, 45].

In addition, the adherence rate was very high, and participants reported enjoyment during play sessions. We assume that the prototype games provided engagement, fun, and motivation for the participants. Interest and enjoyment provided in a game-based environment was pointed out by many researchers [29, 46, 47]. Regarding to play duration, the time dedicated to play *WatchOut* game was much less than the playtime of *HitIt* game because they both implicated doing the same movements nevertheless it was sometimes confusing for participants whether to hit or to avoid falling objects. The game score in *HitIt* game was motivating and created competitiveness between the two participants as they were performing the exercises alternately at the same period of time. They were both eager to score more points by hitting more balls. Social interaction between the two participants had an impact on motivation as well. In this respect, the findings of Wu et al. highlighted the importance of social presence for motivating elderly people while exergaming [18, 48].

Though fatigue was daily reported every 5 or six minutes of play, the games were safe especially with the presence of physiotherapist around. The fall of one participant occurred out of the training sessions, and her return to the intervention program had implied two main points. The first point is that games were engaging and interesting. The second point is that games could be used to restore confidence after falls. Older adults minimize their activities and close up on themselves after fall, besides they express fear of falling again [49]. They usually develop “postfall syndrome” when they fall; in other terms, they become very anxious and cannot stand and walk without support [50]. For that reason, the participant needed more support and reassurance. Games may soften the impact of postfall syndrome and help in the reintegration of the faller into his community [51, 52].

The two subjects included in this case study met the criteria that would be accomplished by the future subjects. Conducting this study, we proved that for subjects that meet these criteria, the system and rehabilitative activities facilitated by the prototype games may be suitable and have allowed closely spotting and improving the issues that elderly subjects may face when interacting with the system providing more effective rehabilitation training for fall prevention.

The use of materials that were relatively small in size recommends a wider screen to facilitate the accessibility to elderly people. Besides, expanding the games repertoire by adding games that require movement and gait to work on static and dynamic balance was highly recommended by the physiotherapist for any future work.

## 5. Conclusion

We conducted a first case study in an elderly house where two participants underwent a 5-week intervention. During the training sessions, the included participants played three prototype games using Microsoft Kinect. The objective of the study was to investigate the feasibility of Kinect-based prototype games in a Tunisian elderly house and study its effectiveness on balance and postural control of elderly people. Consequently, aspects such as enjoyment, adherence, and adverse events were monitored. In addition, balance impact was assessed by the Tinetti balance test. In general, the participants reported enjoyment. They attended the majority of the training sessions and no adverse events were noted. Furthermore, there was an improvement in Tinetti balance test scores.

In conclusion, despite the study sample size was very small, the findings suggest that the use of Kinect-based prototype games for improving balance and postural control of elderly people living in a Tunisian elderly house is feasible and safe and can be integrated in fall prevention programs. In future work, it is intended to conduct a randomized controlled study with a larger sample size to further study the effectiveness of these games.

## Figures and Tables

**Table 1:** Participants' characteristics.

	Participant 1	Participant 2
Age (years)	78	72
Sex	Female	Female
History of falls	2	0
Walking aids	Yes	Yes
A-MMSE	22/30	28/30

**Table 2:** Tinetti balance test scores.

	Participant 1		Participant 2	
	Preassessment	Postassessment	Preassessment	Postassessment
Total score	15	19	14	17
Balance score	7	11	6	10
Gait score	8	8	8	7
